# Automated weighing in the stable isotope lab: When less is more

**DOI:** 10.1016/j.mex.2023.102207

**Published:** 2023-05-05

**Authors:** Matheus C. Carvalho

**Affiliations:** a Southern Cross Analytical Research Services, Southern Cross University, Australia; b Centre for Coastal Biogeochemistry, Southern Cross University, Australia

**Keywords:** Lab automation, Robotics, Autoit, Microbalance autosampler, Cobot, *Miau redux*

## Abstract

Automated powder weighing is an elusive goal in scientific laboratories. The main problem is that powders are much more heterogeneous than liquids, making difficult the development of a unified automation solution for their handling. A compromise has been presented with miau, a low-cost, open-source autosampler for microbalance. Miau was demonstrably useful to perform the automated weighing of some powders, as long as the same powder is weighed repeatedly, which is useful for preparing standards to be measured along samples. However, in stable-isotope laboratories it is also necessary to weigh samples, which are often very heterogeneous, and thus not amenable for miau. Here it is demonstrated how miau can be adapted to handle not only standards, but also samples, using the “less is more” philosophy:

• Miau is simplified to perform only the manipulation of weighing capsules, becoming “miau redux”
• Miau redux can be used not only for standards, but for a variety of samples as well
• Miau redux saves 64% of operator time when using a microbalance

## Graphical abstract

**Figure:**
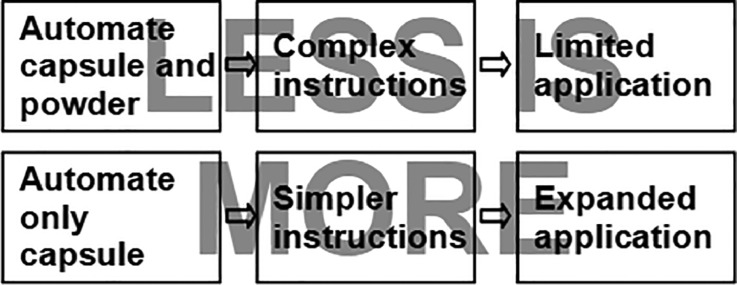

Specifications table

**Table:** 

Subject area:	Chemistry
More specific subject area:	*Stable isotope measurement*
Name of your method:	*Miau redux*
Name and reference of original method:	*M. C. Carvalho, Miau, a microbalance autosampler, HardwareX, 10 (2021), e00215.*https://doi.org/10.1016/j.ohx.2021.e00215
Resource availability:	*Software and 3D print designs supplied in the paper. Firmware and other software in the previous paper.*

## Method details

The method presented here is a modification of a previously published microbalance autosampler, or “miau” [1]. Miau was useful to measure the same substance many times, but not many different substances once or a few times. In a stable isotope laboratory, both activities are important. Standards and quality-control materials are often weighed many times and measured along samples in the instrument in order to monitor measurement accuracy and precision. Automation with this purpose has obvious utility in a stable isotope laboratory, but a large portion of the weighing effort is actually done towards samples which are not measured many times, but only once or maybe two or at most three times for replication. For these cases, the manual procedure is simpler and takes less time.

Miau performed two tasks [1]: 1) manipulation of capsules; 2) powder transfer. Powder transfer is the hard part of the task for miau, as it is for other automated weighing solutions [2]. If, instead of employing miau for these two tasks, it is employed for only the first task, that is, the manipulation of tin capsules, and the second task is performed manually, miau can then be employed for all weighing tasks in an isotope lab, including of samples that are weighed only once. For this new purpose, miau is here called “miau redux”. In other words, miau was “downgraded” to miau redux, and its utility was enhanced, a true case of “less is more”.

It may not be immediately clear why downgrading miau to miau redux is useful for stable isotope measurement. The main reason is that samples for stable isotope analyses of solids can encompass a wide variety of materials, for example, plant and animal tissues (including feathers, which are often not powdered for analysis), mud, sand, rocks, material collected on filters, precipitates, etc. And inside each of these categories there will be variation in terms of grain size, density, texture, propensity for static effects, etc. Another factor is that amounts to be weighed differ according to the kind of sample. For example, while animal tissues and some precipitates can be adequately sampled using masses in the order of 1 mg, sandy sediments will demand 50 mg or more, and filters even more, as they often need to be analyzed whole. In other words, it is arguably impossible to cater for the extreme variation in solid samples in stable isotope investigations. Therefore, instead of attempting to deal with this very difficult problem, miau redux simply helps with the weighing operations that do not involve powder handling, which can be done manually much more easily since these are not so affected by sample heterogeneity.

### Hardware

Miau redux is largely similar to miau, which is described in detail in the original paper. Here just the differences will be explained.

**Fig. 1:**
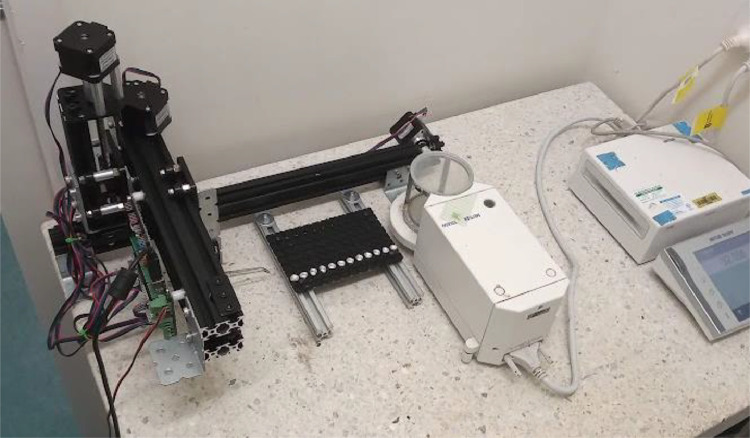
Miau redux close to a microbalance.

Miau redux consists of a cartesian robot with a cantilever structure (Fig. 1).

**Fig. 2:**
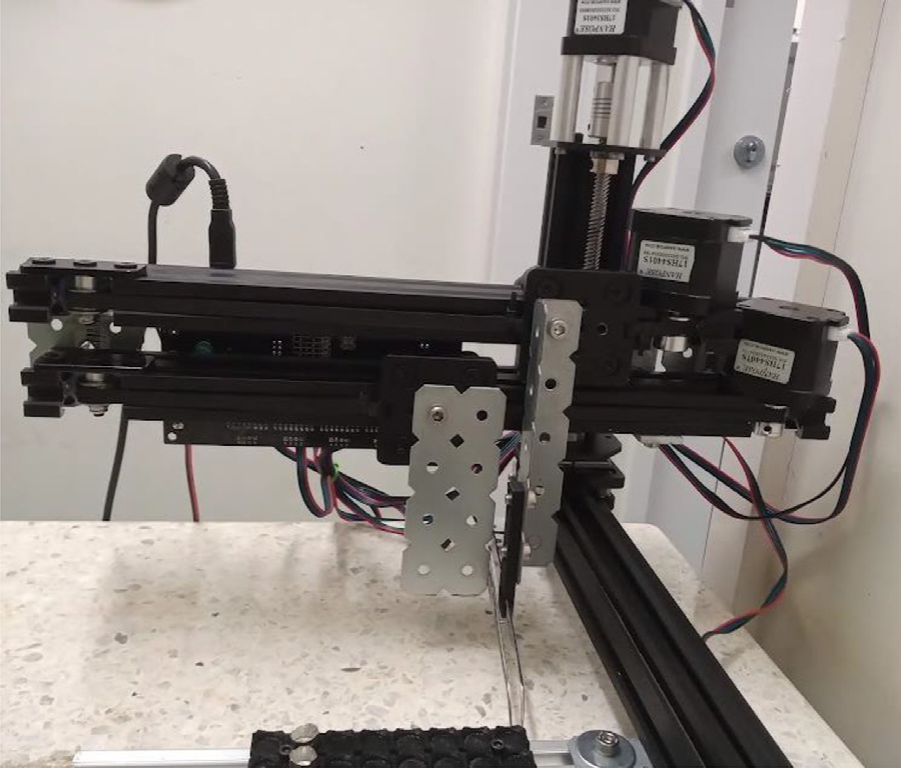
Detail of axes Y (holds forceps) and E (holds bracket). Both are parallel to each other, and are held by the vertical axis (Z).

There is a horizontal axis which supports directly a vertical axis. This vertical axis holds two parallel identical horizontal axes that are perpendicular to the first horizontal axis (Fig. 2). One of the two axes holds a forceps (using a bracket and a 3D-printed support), while the other axis holds a bracket. When both identical axes move at the same time, the forceps moves without changing its aperture (if it is closed, it will remain closed, and if it is open, it will remain open). In order to close the forceps, the two axes are slightly moved in opposite directions, and in order to open the forceps, the opposite procedure is applied, that is, both axes are moved in opposite directions.

Attached to the lower horizontal axis is a 3D-printed sample tray with 96 positions (Fig. 1). This tray is designed to hold tin capsules with 9 mm of diameter and 6 mm on height (e. g. Sercon SC9428).

All motors are controlled using an MKS GEN board, commonly employed to control 3D printers (placed at the back of axes Y and E, Figs. 1 and 2). The firmware is Marlin, also widely employed for 3D printers.

### Software

Miau redux needs to be used with a microbalance that can be controlled using a computer. An AutoIt script [3] is employed to integrate miau redux and the microbalance, and to automatically record the balance readings using spreadsheet software. Differently from the original miau, the weighing process for miau redux is not interactive, that is, the balance readings are not used to evaluate if the weighing process is proceeding correctly or not, which greatly simplifies the software. Because powder transfers are not performed by miau redux, there is not much gain with an interactive procedure. The full source code and explanations about the AutoIt script to control miau redux are in supplementary material 1.

### Installation

**Fig. 3:**
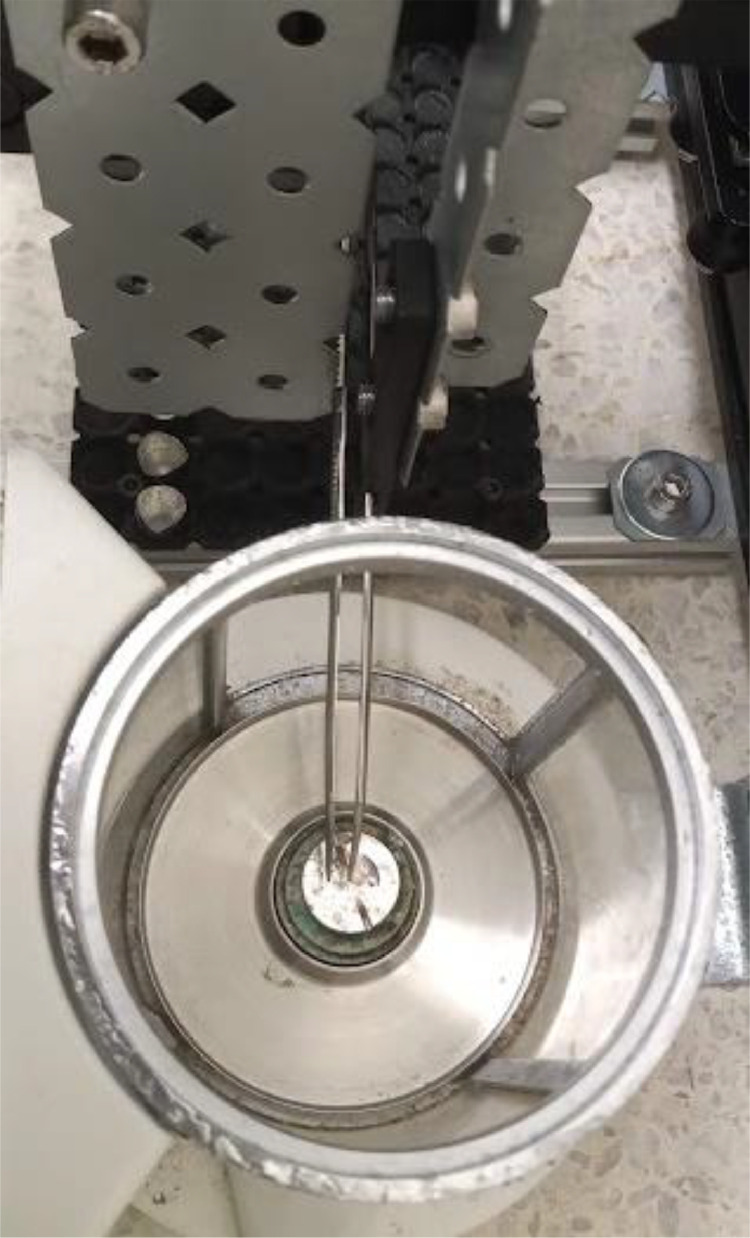
Miau redux forceps inside the balance chamber.

Miau redux needs to be placed close to the microbalance, on top of the weighing table (Fig. 1). These balances are normally equipped with a weighing chamber. The forceps need to be able to go inside the chamber and deliver and remove the capsule from it (Fig. 3).

**Fig. 4:**
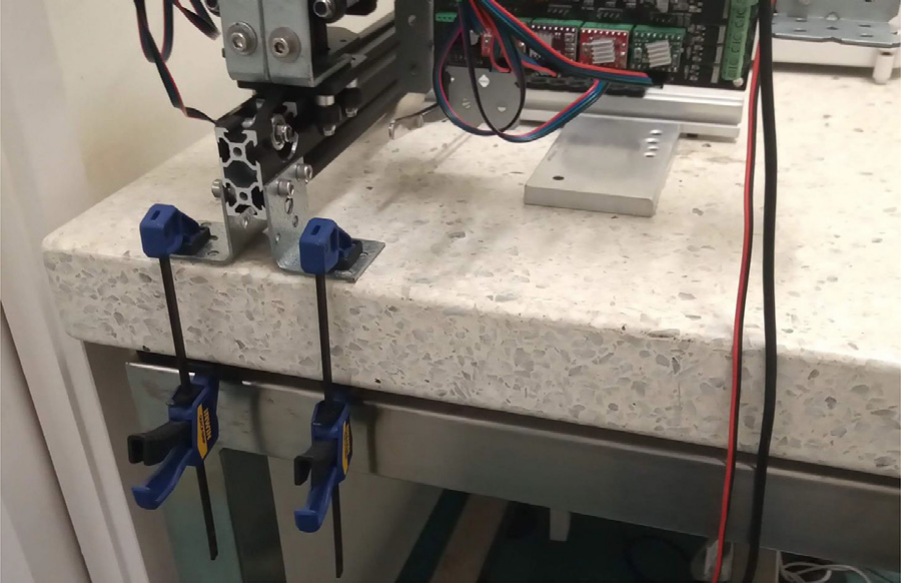
Clamps holding miau redux in place.

It is also important that miau redux is securely attached to the weighing table, otherwise it can slowly drift from its original position due to vibration. Clamps are useful for this (Fig. 4).

### Operation

The typical operation of miau redux consists of: 1) manually placing the empty capsules on the tray; 2) starting miau redux for weighing the empty capsules; 3) manually adding the powders to the capsules on the tray; 4) starting miau redux again, this time to weigh the full capsules; 5) manually wrapping the capsules A video demonstrating the operation is in Supplementary material 2.

It is important that the capsules are not deformed and are placed at the appropriate position on the tray at steps 1 and 3. Failure to do so will make miau redux to miss the capsules. User experience helps, and after some familiarity with the procedure, typically more than 90% of the capsules are handled correctly by miau redux. For example, after step 2, it is possible that miau redux delivers the capsules and they tumble. If so, they need to be manually straightened before powder is added. Also, if too much material is added to the capsule (step 3), there is a risk that some of it will fall during the procedure, so it is advisable not to use the procedure if large amounts of sample need to be prepared.

In addition to these manual aspects dealing with capsules, there are some rare problems that can happen during operation. The comparatively less rare problems are those related to cable entanglement, which can result in loss of power or USB connection. This is easily prevented by careful cable installation when assembling the autosampler. Other potential sources of problems are failure of the control board, stepper motor drivers or stepper motor themselves. However, author experience with these components [1,[4], [5], [6] has shown that they are reliable for years with only very rare problems, if any at all.

### Evaluation

Miau redux allows a significant decrease in the amount of time a person spends weighing. Placing 96 tin capsules in the tray takes about 15 min, because this needs to be done with care to ensure proper positioning of the capsules (more consistent shapes from factory would help decrease the time spent here). Transferring powders takes another 5 – 10 min, and wrapping them again 5 – 10 min. Thus, about 35 min of human work. The manual equivalent would be those 35 min plus the time waiting for the balance twice, once for taring, which takes in the model used here (Mettler Toledo X2TU) takes 30 s, and for weighing again, once filled with sample, another 30 s. So, approximately 1 min is spent just waiting for the balance for each sample, which amounts to 96 min. Therefore, using miau redux a person spends approximately 1 hour less of work for 96 samples, or 64% less time. In a typical stable isotope laboratory, annually 10,000 samples and standards can be reasonably estimated to be prepared for measurement. Miau redux would represent a saving of more than 100 h of human work per annum, or 12.5 full working days (assuming 8 h). It is clear that the cost of installation of miau redux is rapidly recovered with the freed working time. Of course, making and installing miau redux demand some skills, and the time spent on these or contracting a specialized person to perform these will reduce the initial financial savings. Still, by being an open-source project, the return on investment is orders of magnitude higher than for normal commercial scientific equipment [7].

It must be understood that using miau redux does not necessarily speeds up the weighing process, because part of the job is manual. What is warranted is that less human labor is needed for the weighing process. Still, miau redux can be used to accelerate the weighing process, if so desired. This is done by manually performing step 3 (see “operation” for step description) for some capsules while miau redux is still performing step 2, and performing step 5 while miau redux still performs step 4.

### Ethics statements

This method did not involve human subjects, animal experiments, or data collected from social media platforms.

## Supplementary material and/or additional information [OPTIONAL]

Additional information

Stable isotope measurements are widely employed in varied fields of science, including ecology, geology, forensics, and food science. In all these fields, solid samples, commonly obtained by drying and grinding the material of interest, are among the most common [8], [9], [10]. Therefore, a typical device in a stable isotope laboratory lab is a microbalance, as samples normally have masses between 1 and 100 mg [8,11]. Weighing is one of the most common activities in such a laboratory, and hundreds of hours of human labor are employed in this activity in each laboratory every year.

Weighing is a time-consuming activity, not only for stable isotope measurement. As such, it is not surprising that its automation has been attempted. However, the overall scenario is that automated weighing is expensive, complicated, and, although desirable, not really essential, as the benefits fail to overcome the complexity of the automation process [2]. Thus, manual weighing remains very common in all sorts of laboratories across all science disciplines which rely on it.

A recent trend in automation is the utilization of collaborative robots (cobots) instead of robots [12], [13], [14]. Usually, cobots do not automate a task in full, but, instead, work together with human workers. Miau redux can be seen as a cobot, as it complements, and does not replace, a human worker.

Supplementary material

Please see after references

## CRediT authorship contribution statement

**Matheus C. Carvalho:** Conceptualization, Data curation, Formal analysis, Funding acquisition, Investigation, Methodology, Project administration, Resources, Software, Supervision, Validation, Visualization, Writing – original draft, Writing – review & editing.

## Declaration of Competing Interest

The authors declare that they have no known competing financial interests or personal relationships that could have appeared to influence the work reported in this paper.

## Data Availability

Data will be made available on request.

## Appendix Supplementary materials

Image, application 1

Image, video 2
