# Supplementary material for: Automated weighing in the stable isotope lab: When less is more
Source: MethodsX. 2023 May 5;10:102207. doi: 10.1016/j.mex.2023.102207 (PMC10193001; doi:10.1016/j.mex.2023.102207)
Supplement: Supplementary file 1 [file mmc1.docx]

*Supplementary material 1*

Here the code to control miau redux is explained. In order for the code to work, copy the code lines to SciTE and run the code using AutoIt. Only copy the portions in Courier New; don’t copy the parts in arial.

The “Weigh” function of this code works with the balance X2TU from Mettler Toledo. For other balances, this function needs to be modified.

The files commMG.au3 and commMG.dll are needed for the correct execution of the code.

The study of the book “Practical Laboratorial Automation Made Easy With AutoIt”, by Matheus Carvalho, Wiley-VCH, 2016, and also of the original miau paper (DOI: 10.1016/j.ohx.2021.e00215), can be helpful to fully understand the code. Extra software, including Hype!terminal and CommMg files can be found in the file repository for that paper.

1) Libraries

#include <Timers.au3>

#include <commMG.au3>

The library Timers is needed for the weighing function. The library commMG is needed to directly control the balance using AutoIt.

2) Opt

Opt("WinTitleMatchMode", 1)

This instruction allows AutoIt to access correctly the windows names when sending commands to the software being controlled, in this case, Hype!terminal (Fig. S1) and Open office Calc.


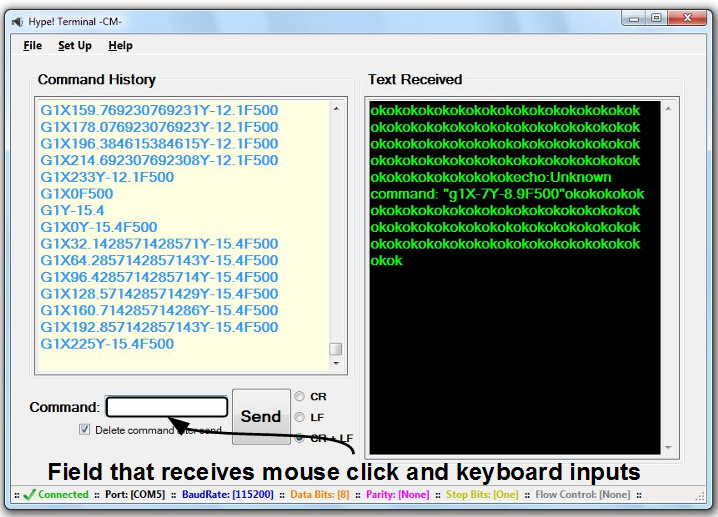


Fig. S1: Hype!terminal interface

3) Variables

$Hypecontrol = "[NAME:tb_cmd]"

Local $setport = 1

Local $sportSetError

Local $BalanceReading

The first variable refers to the text input box in the window Hype!terminal that receives user input. Hype!terminal is the program that sends instructions to miau redux.

The other variables are needed to allow the direct control of the balance.

3) Opening connection with balance

_CommSetPort($setport, $sportSetError, 9600, 8, 0, 1, 2)

The function _CommSetPort uses the variables previously defined to open connection with the balance. The parameters in the function depend on the balance being used.

4) Important positions

*;important positions*

$xs = 0.7

$xe = 5.6

$xn = 12

$ys = 0.05

$ye = 3.4

$yn = 8

$ztin = 6

The first 7 variables ($xs to $ztin) refer to the sample tray. The start positions have an s, the end positions an e. The number of cells on a row or column are indicated by n.

$eopen = 0

$eclosed = 0.05

$yopen = 0

$yclosed = 0.05

The next 4 variables refer to the control of the forceps. Here, the forceps are carried on the Y axis, while the E axis pushes against the Y axis to close the forceps by the offset amount shown by the “closed variables”. To open it again, both axes are moved away of each other using the “open” offsets.

$zsafe = 21

This variable is for the safe Z position for movement. Here, the forceps do not hit the tray or the balance, if the chamber is open.

$xbalance = 9

$ybalance = 2

$zbalance = 16

These variables refer to the position where the forceps place the tin capsule on the balance dish.

5) Array for sample tray

Dim $posxy[200][2]

$n = 0

For $y = 0 To $yn - 1

For $x = 0 To $xn - 1

$posxy[$n][0] = $xs + $x * ($xe - $xs) / ($xn - 1)

$posxy[$n][1] = $ys + $y * ($ye - $ys) / ($yn - 1)

$n = $n + 1

Next

Next

The array $posxy is created using the 6 initial variables. Notice that it takes more disk space (200) than necessary. If a different tray with more than 200 positions on a single direction is used, this need to be modified.

6) Functions for controlling the robot

*;basic moves*

Func Move($direction, $position, $speed, $wait_time, $control)

ControlSetText("Hype!", "", $control, "G1" & $direction & $position & "F" & $speed)

Sleep(100)

ControlSend("Hype!", "", $control, "{ENTER}")

Sleep($wait_time)

EndFunc *;==>Move*

Func MoveXYE($position1, $position2, $position3, $speed, $wait_time, $control)

ControlSetText("Hype!", "", $control, "G1X" & $position1 & "Y" & $position2 & "E" & $position3 & "F" & $speed)

Sleep(100)

ControlSend("Hype!", "", $control, "{ENTER}")

Sleep($wait_time)

EndFunc *;==>MoveXYE*

Func MoveYE($position1, $position2, $speed, $wait_time, $control)

ControlSetText("Hype!", "", $control, "G1Y" & $position1 & "E" & $position2 & "F" & $speed)

Sleep(100)

ControlSend("Hype!", "", $control, "{ENTER}")

Sleep($wait_time)

EndFunc *;==>MoveYE*

These three functions control the robot via Hype!terminal. Each of them receives parameters and sends a G-code command to Hype!terminal. The first function moves a single axis at once. The second function moves the axes X, Y and E simultaneously, while the third function moves the axes Y and E simultaneously.

7) Other functions

*;Balance functions*

Func GetNumberFromArray($Array)

$mass = -2

For $i = 0 To UBound($Array) - 1

If $Array[$i] - 1 <> -1 Then

$mass = $Array[$i]

EndIf

Next

Return $mass

EndFunc *;==>GetNumberFromArray*

Func Weigh()

Dim $ReadingArray[10] = [-1, -1, -1, -1, -1, -1, -1, -1, -1, -1]

_CommSendString("SU" & @CR)

Sleep(10 * 521)

$StartBal = _Timer_Init()

$BalanceReading = _CommReadString(10 * 60 * 521) *;problem here*

$TimeSpent = _Timer_Diff($StartBal)

If $TimeSpent > 5 * 60 * 521 Then Exit

Sleep(230)

$ReadingArray = StringSplit($BalanceReading, " ")

$mass = GetNumberFromArray($ReadingArray)

Sleep(230)

Return $mass

_CommSendString("WS 2" & @CR)

EndFunc *;==>Weigh*

*;Processing functions*

Func WriteToOO($sample, $mass, $column)

WinActivate("AutoWeigh")

Sleep(230)

Send("^[15]")

Sleep(230)

For $i = 1 To $column

Sleep(100)

Send("{RIGHT}")

Next

For $i = 1 To $sample

Sleep(100)

Send("{DOWN}")

Next

Sleep(230)

Send($mass & "{ENTER}")

Send("^[15]")

Sleep(230)

Send("^{s}")

EndFunc *;==>WriteToOO*

These functions are fully explained in the original miau paper.

8) Main code

For $sample = 0 To 95

Move("Z", $zsafe, 397, 007, $Hypecontrol)

MoveXYE($posxy[$sample][0], $posxy[$sample][1] + $yopen, $posxy[$sample][1] + $eopen, 521, 007, $Hypecontrol)

Move("Z", $ztin, 998, 007, $Hypecontrol)

MoveYE($posxy[$sample][1] + $yclosed, $posxy[$sample][1] + $eclosed, 521, 007, $Hypecontrol)

Move("Z", $zsafe, 397, 007, $Hypecontrol)

MoveXYE($xbalance - 4, $ybalance + $yclosed, $ybalance + $eclosed, 521, 007, $Hypecontrol)

Move("X", $xbalance, 521, 007, $Hypecontrol)

Move("Z", $zbalance, 998, 007, $Hypecontrol)

MoveYE($ybalance + $yopen, $ybalance + $eopen, 521, 007, $Hypecontrol)

Move("Z", $zsafe, 397, 007, $Hypecontrol)

Move("X", 0, 521, 007, $Hypecontrol)

Move("Z", 0, 998, 007, $Hypecontrol)

Sleep(20000)

$mass = Weigh()

WriteToOO($sample+1, $mass, 1)

Move("Z", $zsafe, 397, 007, $Hypecontrol)

Move("X", $xbalance, 521, 007, $Hypecontrol)

Move("Z", $zbalance - 0.4, 998, 007, $Hypecontrol)

MoveYE($ybalance + $yclosed, $ybalance + $eclosed, 521, 007, $Hypecontrol)

Move("Z", $zsafe, 397, 007, $Hypecontrol)

Move("X", $xbalance - 4, 521, 007, $Hypecontrol)

MoveXYE($posxy[$sample][0], $posxy[$sample][1] + $yclosed, $posxy[$sample][1] + $eclosed, 521, 007, $Hypecontrol)

Move("Z", $ztin + 0.5, 998, 007, $Hypecontrol)

MoveYE($posxy[$sample][1] + $yopen, $posxy[$sample][1] + $eopen, 521, 007, $Hypecontrol)

Move("Z", $zsafe, 397, 007, $Hypecontrol)

Next

MoveXYE(0, 0, 0, 521, 007, $Hypecontrol)

Move("Z", 0, 998, 007, $Hypecontrol)

The main function of the code is a loop. It starts at 0 and goes to 95, if 96 samples are to be weighed. In order to be used correctly, it is important that the forceps are resting at their original position, that is, all axes equal to zero. At this position, the forceps are lowered at the minimum possible height at the vertical (Z) axis, it is placed at the maximum possible distance from the balance on the X axis, the Y axis is at its most retracted position, and the E axis is at a position which leaves the forceps with a 3 mm aperture (Figs S2 and S3).


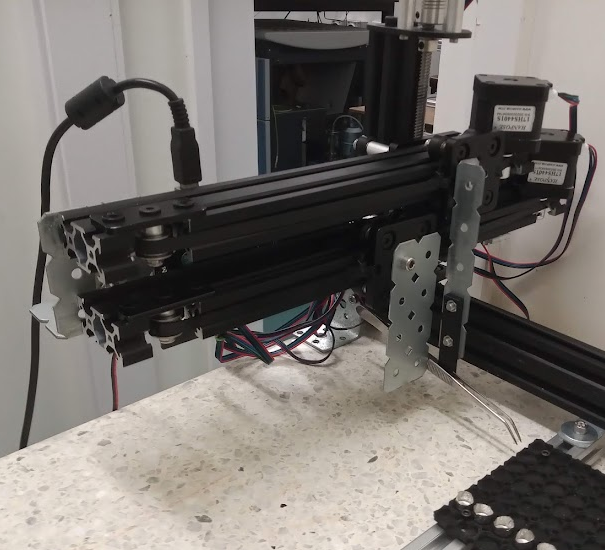


Fig. S2: Zero position.


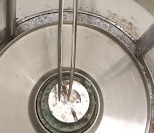


Fig. S3: Forceps aperture, about 3 mm.

The first movement is to bring the forceps to $Zsafe, because it starts at Z = 0, that is down. When at the safe position, the forceps are moved to the position of the first sample. Notice that Y and E move together. The third movement is to bring the forceps down to the tin capsule height. The fourth movement is to close the forceps, grabbing the tin capsule. Movement 5 brings the forceps up, holding the capsule, to the safe position. Movement 6 moves the forceps to a position facing the balance dish, but outside the balance chamber. Movement 7 penetrates the chamber with the forceps. Movement 8 lowers the forceps to the appropriate height to release the tin capsule on the balance dish. Movement 9 releases the capsule on the balance dish. Movement 10 rises the forceps to safe position. Movement 11 brings the forceps to position X = 0, that is, the origin of the X axis. Movement 12 lowers the forceps to position Z = 0, which is a resting position. Then there is a waiting time of 20 seconds, after which the balance is activated using the function Weigh. Once the weigh is registered, the value is sent to an open office spreadsheet that has a file open starting with the name “AutoWeigh”. The third parameter, shown with the number 1, is the first column on the spreadsheet. It needs to be modified for the second use of the robot, when the filled capsules are weighed, otherwise these values will be overwritten. Once the weighing value is registered on the spreadsheet software, miau redux does is 13^th^ movement, which is to bring the forceps up to the safe position. Movement 14 brings the forceps inside the balance chamber. Movement 15 lowers the forceps to the capsule height. Movement 16 closes the forceps, grabbing the capsule. Movement 17 rises the forceps and the capsule to the safe position. Movement 18 removes the forceps from the balance chamber. Movement 19 takes the forceps to the capsule original position on the tray. Movement 20 lowers the capsule to close to the tray. Movement 21 releases the capsule on the tray. Movement 22 rises the forceps to safe position.

The procedure above repeats for all samples. Once all of them are finished, there are two more movements: the forceps are brought to position X = Y = E = 0, and then Z = 0.

*Supplementary material 2*

Video

*Supplementary material 3*

The drawings below are made using OpenScad, a program which takes written instructions to generate 3D designs. These designs need to be exported to STL files, which then can be sent to a 3D printer program to be printed.

Forceps holder


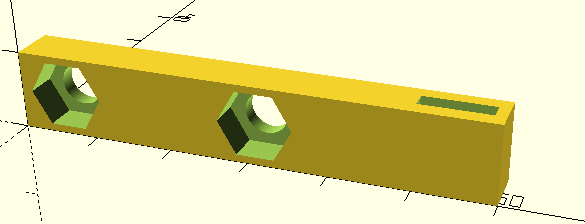


bar1x =60;//horizontal bar

bar1y = 6;

bar1z = 10;

bar1tx = 0;

bar1ty = 0;

bar1tz = 0;

hole1r = 2.7;//5mm hole

hole1z = bar1z;

hole1tx = bar1tx+6;

hole1ty = bar1ty+10;

hole1tz = bar1tz+bar1z/2;

hole2r = 4.7;//5mm hole

hole2z = 4;

hole2tx = hole1tx;

hole2ty = hole1ty-6;

hole2tz = hole1tz;

forcepsx = 8.7;//space for forceps

forcepsy = 2.2;

forcepsz = 50;

forcepstx = bar1tx+bar1x-12;

forcepsty = bar1ty+2;

forcepstz = bar1tz;

$fn=64;

difference(){

translate([bar1tx,bar1ty,bar1tz]){cube([bar1x,bar1y,bar1z]);}

#translate([hole1tx,hole1ty,hole1tz]){rotate([90,0,0]){cylinder( hole1z, hole1r, hole1r, false);}}

#translate([hole2tx,hole2ty,hole2tz]){rotate([90,0,0]){cylinder( hole2z, hole2r, hole2r, false,$fn=6);}}

#translate([hole1tx+25,hole1ty,hole1tz]){rotate([90,0,0]){cylinder( hole1z, hole1r, hole1r, false);}}

#translate([hole2tx+25,hole2ty,hole2tz]){rotate([90,0,0]){cylinder( hole2z, hole2r, hole2r, false,$fn=6);}}

#translate([forcepstx,forcepsty,forcepstz]){rotate([0,10,0]){cube([forcepsx,forcepsy,forcepsz]);}}//forceps

}

Sample tray


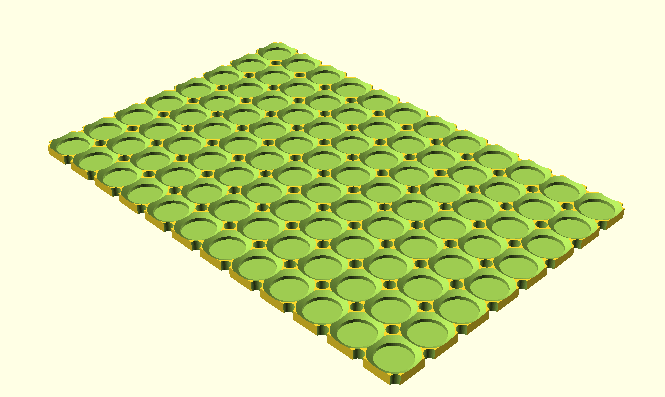


cellx=12;

celly=12;

cellz=3;

module TinHole(){

holer1 = 5.0;

holer2 = 6.6;

holez = 1;

translate([0,0,0]){cylinder(holez,holer1,holer1,false);}

translate([0,0,holez]){cylinder(1,holer1,holer2,false);}

}

module Cell(){

screwr=1.7;

difference(){

translate([0,0,0]){cube([cellx,celly,cellz]);}

#translate([cellx/2,celly/2,1]){TinHole();}

#translate([cellx,celly,0]){cylinder(10,screwr,screwr,false);}

#translate([0,celly,0]){cylinder(10,screwr,screwr,false);}

#translate([cellx,0,0]){cylinder(10,screwr,screwr,false);}

#translate([0,0,0]){cylinder(10,screwr,screwr,false);}

}

}

$fn=64;

xn=11;

yn=7;

for(x=[0:cellx:cellx*xn]){

for(y=[0:celly:celly*yn]){

translate([x,y,0]){Cell();}

}

}
